# Supplementary material for: Wa-1 Equine-Like G3P[8] Rotavirus from a Child with Diarrhea in Colombia
Source: Viruses. 2021 Jun 4;13(6):1075. doi: 10.3390/v13061075 (PMC8226935; doi:10.3390/v13061075)
Supplement: Supplementary file 1 [file viruses-13-01075-s001.zip › viruses-1210483-supplementary.pdf]

viruses

MDPI

Supplementary material

Wa-1 equine-like G3P[8] rotavirus from a child with diarrhea in Colombia

Table S1. List of Primers used for RVA amplification and sequencing [(1)].

**VP1 (a+b+c)**		**VP1a (1-1164)**
	VP1-F-04	GGC TAT TAA AGC TRT AC
	VP1-2-R	CTT GAA GAA GAT GAT CAT CAT GC
		VP1b (1142-2786)
	RV-VP1bF	TGA AGA CAG AGT ACA CTG AAG ATG
	RV-VP1bR	ATC CAG TAA TGG CTA CAG ATG TC
		VP1c (2461- 3288)
	VP1-F-02	GAG GAC AAA GTA CAC AAT GGG
	VP1-R-04	GGT CAC ATC TAA GCG
**VP3 (a+b+c)**		**VP3a (1 -1083)**
	VP3-F	GGC TWT TAA AGC AGT ACY
	VP3a-R	CCA CTC TTT CCA ATC CAT AT
		**VP3b (714-1797)**
	VP3b-F	TAT TAA ATT GAA GCA GGA GAA ATG G
	VP3b-R	ACA TTA GTA CCT ATT AGA TTG AA
		**VP3c (1410-2573)**
	VP3-F-In06	GAT TAT ATC GTA GCA TTA TAC GC
	GEN-VP3R	GGY CAC ATC ATG ACT AGT G
**VP4 (a+b)**		**VP4a (1-1193)**
	VP4-1-a	GGC TAT AAA ATG GCT TCG CTC ATT
	VP4a-R	AAA CAG CGC CAC CAT TCA TTA
		**VP4b (1082-2338)**
	VP4b-F	TGG GAT GAT TCA AAA GCA TT
	VP4b-R	GGT CAC ATC CTC AAT AGC GTT C
**VP6 (1-1340)**	GEN-VP6F	GGC TTT WAA ACG AAG TCT TC
	GEN-VP6R	GGT CAC ATC CTC TCA CT
**VP7 (1-1036)**	Beg	GGC TTT AAA AGA GAG AAT TTC CGT CTG G
	End	GGT CAC ATC ATA CAA TTC TAA TCT AAG
**NSP1 (1-1599)**	NSP1-F	GGC TTT TTT TTG AAA AGT CTT GTG
	NSP1-R	GGT TCA CAG TTT TTG CTG GCT AGG
**NSP2 (1-1042)**	GEN-NSP2F	GGC TTT TAA AGC GTC TCA G
	GEN-NSP2R	GGT CAC ATA AGC GCT TTC
**NSP3 (1-1050)**	GEN-NSP3F	GGC TTT TAA TCG TTT TCA GTG
	NSP3-R	GGT CAC ATA ACG CC CTA TAG C
**NSP4 (3-741)**	NSP4-1a	GGC TTT TAA AAG TTC TGT TCC G
	NSP4-R	GGT CAC ATT AAG ACC ATT CCT TCC
**NSP5 (1-663)**	NSP5-Beg	GGC TTT TAA AGC GCT ACA GTG ATG
	NSP5-End	GGT CAC AAA ACG GGA GTG GG

[1]Primers from: Degiuseppe JI et al., Clin Microbiol Infect. 2013;19(8): E367-71.
